# Towards the Identification of Antibiotic-Resistant Bacteria Causing Urinary Tract Infections Using Volatile Organic Compounds Analysis—A Pilot Study

**DOI:** 10.3390/antibiotics9110797

**Published:** 2020-11-11

**Authors:** Keith Hewett, Natalia Drabińska, Paul White, Matthew B. Avison, Raj Persad, Norman Ratcliffe, Ben de Lacy Costello

**Affiliations:** 1 Department of Applied Sciences, Faculty of Health and Applied Sciences, University of the West of England, Coldharbour Lane, Bristol BS16 1QY, UK; Keith2.Hewett@uwe.ac.uk (K.H.); Norman.Ratcliffe@uwe.ac.uk (N.R.); 2 Department of Chemistry and Biodynamics of Food, Institute of Animal Reproduction and Food Research of Polish Academy of Sciences, 10-748 Olsztyn, Poland; 3 Applied Statistics Group, Department of Engineering, Design and Mathematics, Faculty of Environment and Technology, University of the West of England, Bristol BS16 1QY, UK; Paul.White@uwe.ac.uk; 4 School of Cellular & Molecular Medicine, Faculty of Life Sciences, University of Bristol, Bristol BS8 1TD, UK; bimba@bris.ac.uk; 5 Bristol Royal Infirmary and Bristol Urological Institute, Southmead Hospital, Bristol BS10 5BN, UK; rajpersad@bristolurology.com

**Keywords:** volatile, metabolite, profiles, gas chromatography-mass spectrometry, antibiotic resistance, bacteria, urinary tract infection, susceptibility, *E. coli*

## Abstract

Antibiotic resistance is an unprecedented threat to modern medicine. The analysis of volatile organic compounds (VOCs) from bacteria potentially offers a rapid way to determine antibiotic susceptibility in bacteria. This study aimed to find the optimal conditions to obtain the maximum number of VOCs detected which next allowed the assessment of differences in VOC profiles between susceptible and resistant isolates of *Escherichia coli* causing urinary tract infections. The analysis of VOCs in the headspace above the bacterial cultures allowed the distinguishing of resistant and susceptible bacteria based on the abundance of six VOCs with 85.7% overall accuracy. The results of this preliminary study are promising, and with development could lead to a practical, faster diagnostic method for use in routine microbiology.

## 1. Introduction

There is a need for more rapid determination of antibiotic resistance (ABR) in bacteria isolated from clinical samples. ABR is becoming more prevalent, as are urinary tract infections (UTIs) due to an aging population. Community-acquired UTIs have a prevalence of 0.7% in the United States corresponding to 7–8 million infections per year. This rises to 4% amongst healthcare-associated infections in the USA and 6% in Europe [1]. In healthcare-associated infections, the proportion of isolates resistant to specific antibiotics is rarely below 10% and in the case of ampicillin is above 40% [2]. Rapid susceptibility testing and appropriate treatment will decrease morbidity and healthcare costs, plus protect the efficacy of broad spectrum agents.

With the discovery of the first antibiotic came the discovery of the first mechanism of resistance, in *Escherichia coli*, now known as the β-lactamase AmpC [3]. As the use of penicillin became widespread, so did plasmid encoded penicillinases, first in *Staphylococcus aureus*, followed by other staphylococci. In the 1960s the first plasmid mediated β-lactamase in Gram-negatives was described [4]. The enzyme was given the designation TEM from the name of the patient from whom the strain was first isolated (Temoniera), and within a few years had spread worldwide. This was accompanied by the emergence of the related sulphydryl variable (SHV) and oxacillinase subgroups [4]. In the last few decades, numerous second, third and fourth generation cephalosporin antibiotics have been developed, and yet further extended spectrum β-lactamases (ESBLs) have emerged that confer resistance in many organisms. From the late 1990s onwards, the TEM and SHV ESBL enzyme variants most commonly associated with nosocomial infection began to be outnumbered by CTX-M enzymes. These enzymes are named after their ability to efficiently hydrolyse the third generation cephalosporin cefotaxime (the M coming from Munich, the city of first isolations). They appear to be transmitted more readily to a broader range of Enterobacteriales, including many strains of the *Escherichia coli*. It is for this reason that CTX-M enzymes are no longer associated with only nosocomial infections and have become associated with many more “epidemic” clones affecting multiple sites [5]. Since the turn of the millennium there has been an significant rise in CTX-M-associated infections leading some to describe the situation as a pandemic [6]. Further to this, there has been an emergence of *E. coli* strains hyperproducing the chromosomally encoded cephalosporinase AmpC and plasmid encoded AmpC-type cephalosporinases, which are different from ESBLs in that they are not inhibited by the β-lactamase inhibitor clavulanic acid [7]. In view of these facts, prompt identification and identification of ESBL-producing or AmpC-expressing bacteria is of crucial importance.

The aim of many rapid diagnostics is to confirm infection and then susceptibility. Species identification is not always necessary but can allow predictions about susceptibility in the absence of any other test, and might help suggest source of infection, or likely prognosis. Identification of pathogens and their resistance profile is still performed by traditional methods of culture and sensitivity in the majority of routine laboratories worldwide. The typical time to confirm culture positivity, bacterial identification and susceptibility is 24–72 h from urine, and 72 h from blood. Some automated or semiautomated systems such as the Phoenix (BD Diagnostic Systems, Heidelberg, Germany) or the Vitek 2 (bioMérieux, Marcy-l’Étoile, France) can speed up the sensitivity testing, but the primary culture is still required. In the last 20 years, there has been a drive to reduce this timespan, mainly by focussing on detection of known resistance genes. Starting with gene-specific PCR assays [8,9] and later DNA microarrays [10], DNA based techniques have the advantage of identifying specific sequences relating to resistance, and are useful in the investigation and evolution of resistance profiles. However, relevant sequences must be studied beforehand, and the costs and skills associated with these techniques puts them out of reach of routine laboratories. Alternatively, matrix-assisted laser desorption/ionisation time of flight mass spectrometry (MALDI-TOF-MS) can be used to investigate the proteome of organisms. In this technique, microbial samples are immobilised in a matrix before ionisation by laser. Mainly ribosomal proteins in the 2–20 kDa range are ionised and quantified by time of flight mass spectrometry, and the resulting peptide mass fingerprint is used to identify specific organisms. More recent work has shown that MALDI-TOF-MS may have some potential in the rapid indication of antimicrobial resistance [11,12,13]. However, whilst MALDI-TOF mass spectrometers have found their way into some routine laboratories, their cost is still prohibitive for most, and many resistance proteins are larger than the 20 kDa limit.

The potential for volatile organic compound (VOC) analysis as a means of identifying and characterising antimicrobial resistance has shown great promise to this group and others [14,15], and this technique may have advantages over other rapid methods. Following on from success in identifying susceptible and resistant strains of a number of organisms by volatile profile [16] it was thought that distinguishing between ESBL positive or negative strains of *E. coli* may be possible, enabling further development of screening methods for patients, potentially by direct sampling of their urine on or after admission. This preliminary study aimed to distinguish the most common and problematic ESBL variant seen in the UK, namely CTX-M-15, from susceptible strains of the same organism, in broth culture.

In order to establish whether ESBL strains could be distinguished from other strains of *E. coli* it was important to devise a methodology that was both standardised enough to observe any significant differences and simple enough to be performed in a routine laboratory. The simplest and most commonly performed method of analysing volatiles produced by microorganisms is analysis of the headspace above a vial of bacteria in planktonic culture. In an ideal situation, this would be performed directly from the vial into a suitable instrument such as a gas chromatograph-mass spectrometer (GC-MS). However, as such instruments are not currently available in routine microbiology laboratories a method for sampling remotely and transporting between sites was required. Solid-phase microextraction (SPME) fibres have gained popularity in recent years as sample extraction and preconcentration is achieved in a single step. However, when studying samples of a complex heterogeneous nature, a number of pitfalls must be avoided [17]. The precoated surface of the fibre is exposed to the headspace above liquid cultures of bacteria and over a period of time, VOCs are adsorbed onto the surface until an equilibrium is reached. The nature of this process, however, depends hugely on the composition of the surface, the time given to reach equilibrium and the temperature at which this occurs. During the equilibration, loss of compound can occur by either biodegradation or adsorption to other surfaces. Fibres are also coated with many different coatings and these all adsorb some compounds preferentially to others. Balasubramanian and Panigrahi provide an exhaustive review of these factors and others, and it can be seen that most studies that compare SPME techniques show greater variance between samples than with other techniques [18,19,20]. It was therefore very important for this study that readily available SPME fibres were used and incubation and storage conditions were carefully selected to optimise the competing goals of sample standardisation and repeatability of the method by routine laboratories.

The aim of this study was to find the optimal conditions to obtain the maximum number of VOCs detected in order to distinguish between susceptible and resistant strains of particular organisms and antimicrobials. The best outcome for any potential diagnostic method would be an ability to distinguish between strains of *E. coli* with different resistance profiles by a single sampling of vial headspace by SPME fibre. The first arm of this study therefore compared several replicates of two groups of four strains. One group were CTX-M-15 ESBL strains and the other susceptible strains of *E. coli*. Any discrimination of the two groups by VOC profile alone would be considered highly significant and important. However, if no such discrimination were to be seen, the next best scenario would be the ability to rapidly distinguish between the groups by monitoring the volatile profile over time until divergence was seen. Whilst not as significant as positive results in the first arm, these experiments would also demonstrate a potential diagnostic method.

## 2. Results and Discussion

### 2.1. Selecting of the Optimal Microbiological Conditions

**Figure 1:**
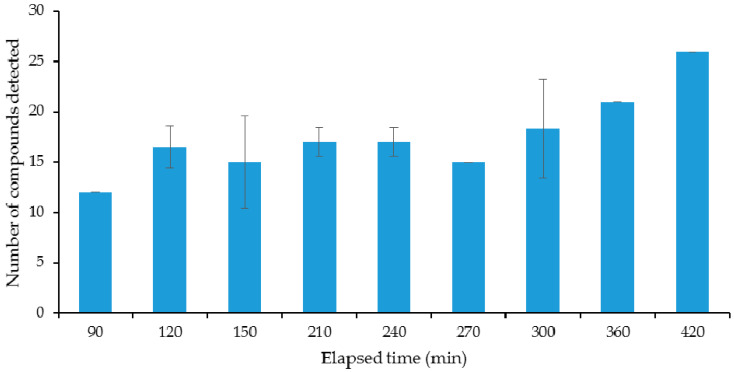
Number of volatile organic compounds (VOCs) detected in the headspace above a laboratory strain of *E. coli* over time.

**Figure 2:**
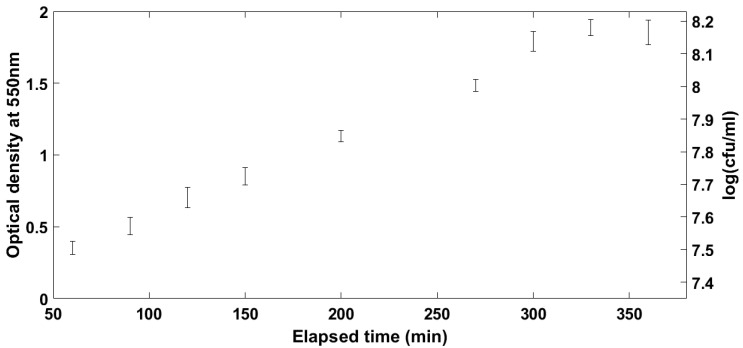
Optical and cell density of laboratory strain of *E. coli* used in preliminary experiments.

**Figure 3:**
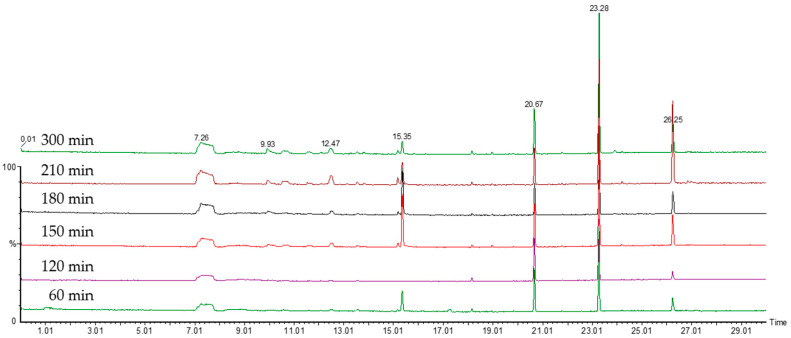
The chromatograms showing the increase of the VOCs with the increasing time of the incubation, up to 300 min.

In order for any measurement of VOCs to be comparable between samples, the point at which the organisms are on their growth profile must be well described and understood. Broth cultures of *E. coli* were sampled over time using the SPME fibres to be used in the main study. For the optimisation of the incubation time, a laboratory DH5α strain of *E. coli* was used as an example organism. Results of the initial experiments on the evolution of the VOCs’ profile over culture time can be seen in Figure 1, Figure 2 and Figure 3. Figure 1 shows the number of different compounds detected at the different time points, Figure 2 shows the optical density and cell density of this strain at these time points, while Figure 3 shows the evolution of VOCs with time. The number of VOCs increased with the increasing incubation time.

The effect of different culture media on the VOC profiles produced by a wide variety of organisms has been well studied [21,22,23]. It is obvious that the substrate available to microorganisms greatly influences the VOCs they are able to produce, but other factors such as pH, salt concentration and temperature also have large effects. Depending on the manner in which the organisms are growing and multiplying, these initial conditions can change considerably as time progresses. In ex vivo samples, microbes are growing in competition with the host environment and although relatively steady states are common there are many confounding sources of VOCs. These include those produced by inflammatory host response [24], exogenous factors [25] and commensal organisms [26]. In vitro studies avoid these confounding factors, but introduce problems of their own. Most of these studies rely on headspace analysis of bacteria in planktonic culture. In the simplest case of a low cell count of a single strain of a microorganism inoculated into a small volume of liquid media, the organism will first pass though the lag phase. This is a distinct growth phase where the organism is acclimatising to the conditions into which it has been introduced and preparing for the next stage [27]. Next, the organism enters the log, or exponential growth phase. At first, the easiest to metabolise energy sources will be consumed [28] but as these substrates are depleted, hydrolytic pathways are induced [29] and catabolite repression is lifted [30]. It is during this phase that the number and quantity of VOCs produced increases. In fact, it has been shown that *E. coli* grown in batch culture in Luria−Bertani (LB) medium passes through four distinct phases in the log phase and each is likely to produce a different VOC profile [31]. It is likely that it is during this phase that the optimum time for volatile sampling is reached. As nutrients run out completely, the organism enters the stationary phase.

**Figure 4:**
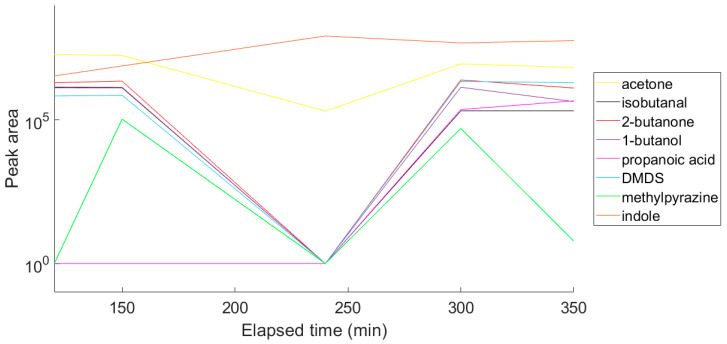
Variation in concentration of nine VOCs over time as a laboratory strain of *E. coli* grown in broth culture.

**Figure 5:**
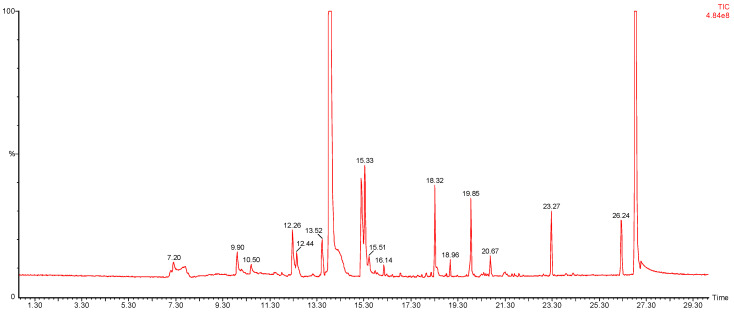
A representative chromatogram of *E. coli* bacterial culture, using the SPME fibre preconcentration method, and GC-MS.

Next, nine VOCs representing different chemical groups and the compound characteristics for *E. coli* [32,33] were selected and the variation in the abundance of these compounds over time was followed (Figure 4). A time course was performed to enable selection of a suitable time point for a diagnostic test. For this reason, the optimum sampling point was chosen to be at an optical density of approximately 1.6 at 550 nm. The representative chromatogram of the optimised conditions is shown in Figure 5, and the list of the tentatively identified VOCs is presented in Table 1.

### 2.2. Comparison of VOC Profiles of Resistant and Susceptible Bacteria

In order to verify the applicability of this method for distinguishing resistant and susceptible bacteria, the VOC profiles were detected at the optimised time point. The comparison study was performed based on the true replicates of organisms from different primary cultures on different days. In total there were 84 samples comprising 45 susceptible and 39 resistant isolates, allowing for multiple isolates of the 8 strains. Across all 84 samples a total of 85 VOCs (Table 2) were detected although some occurred very infrequently. Some 30 VOCs were detected in the headspace above the LB medium, used for bacterial culture, and this information was included in further analysis. The VOCs detected in the headspace above the bacterial cultures were characteristic for the *E. coli* species, with a predominant VOC being indole [32,33]. Bacteria produce a wide and diverse range of VOCs. These compounds may be produced as byproducts of metabolism, but they can also act as signalling molecules for communication between bacteria, or between bacteria and host. Although these interactions are not fully understood, they are thought to play an important role in antibiotic resistance, as described in a review by Bos et al. [34].

**Figure 6:**
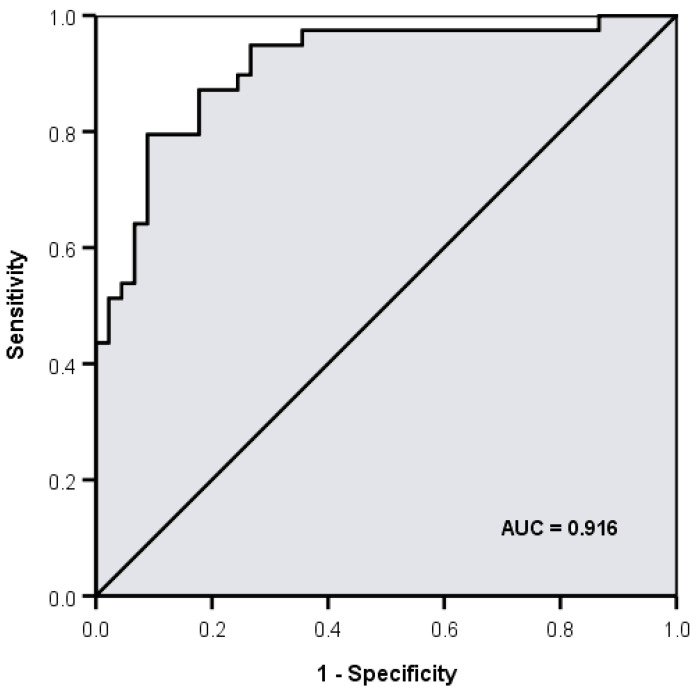
Area under the receiver operating characteristic curve (AUROC) for discrimination between extended spectrum β-lactamase (ESBL) positive and negative strains using 6 volatiles (retention times (min) 16.68, 18.5, 19.47, 20.40, 24.36 and 25.02) = 0.912.

**Figure 7:**
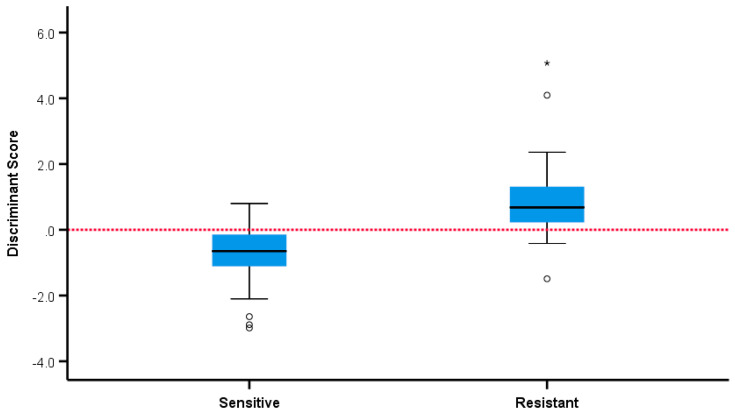
Separation of sensitive and ESBL positive and negative strains by discriminant score. * —outliers.

The statistical analysis showed that five volatiles (retention times (RT) expressed in min: 16.68, 18.5, 19.2, 19.47, 20.40) were positively associated with the samples from resistant strains with *p* < 0.05 in all five cases (*p* = 0.039, *p* = 0.011, *p* = 0.024, *p* = 0.030, *p* = 0.003, respectively). Two volatiles (RT: 24.36, 25.07) were positively associated with the samples from sensitive strains (*p* = 0.005, *p* = 0.045, respectively). In a multivariate analysis, the six volatiles with RT (mins): 16.68, 18.5, 19.47, 20.40, 24.36, 25.07 independently and jointly contributed to overall discrimination between susceptible and resistant strains with 85.7% overall accuracy, with almost the same accuracy under leave-one-out cross-validation. A total of 91.1% of samples from sensitive strains and 79.5% of samples from resistant strains were correctly classified and discriminant scores are summarised in Figure 6 and Figure 7. The microbiological methods used here involved true replicates of organisms from different primary cultures on different days, leading to a relatively large variance between samples. The fact that a significant discrimination between the groups (AUROC 0.912) could be seen using only six volatiles is extremely encouraging.

Unfortunately, exact identification of the VOCs discriminating the susceptible and resistant bacteria was not possible, except for the tentative identification of butanoic acid and 2-dodecanone. The mass spectra of the other four VOCs are presented in the Figure S1. The abundance of butanoic acid was higher in the resistant bacteria, while the abundance of 2-dodecanone was higher in the resistant strains. Butanoic acid is the product of bacterial fermentation [35]. Interestingly, 2-dodecanone was detected in *E. coli* strains after the incubation with biosilver nanoparticles, having bacteriostatic and bactericidal properties.

The results of this preliminary study are promising, and with development could lead to a practical diagnostic method for use in routine microbiology to distinguish between strains that do or do not carry ESBL. Currently, organisms are incubated for 24 h in a primary broth before transfer to a second standardised broth. However, infected urine typically contains greater than 10^5^ cfu/mL of bacterial cells, this being two log fold below the optimum sampling point seen. If the aim is to rapidly screen for ESBL strains present in patient urine, then it is conceivable that this could be possible by enriching with broth and incubating the samples directly. In order to do this, it must be accepted that better characterisation of the discriminating volatiles is necessary.

The method of VOC detection by GC-MS has successfully identified important volatiles by retention time, but full identification has not been possible other than a chemical group (mostly alkanes). If further identification can be achieved, tailoring of the culture or enrichment media can be performed, promoting production of the VOCs of interest, thus increasing the sensitivity of the technique. It is also possible that simpler and cheaper methods of detecting the volatiles could be employed, such as those based around electrochemical sensing.

## 3. Materials and Methods

### 3.1. Bacterial Cultures and Sampling

To first establish the protocol for VOC sampling, 250 mL of Luria−Bertani (LB) broth was inoculated with a laboratory strain of *E. coli* and incubated overnight at 37 °C. This was then subcultured into a bank of headspace vials containing 3 mL of LB at a ratio of 20 μl/mL. These were reincubated at 37 °C with shaking. At 30 min intervals, SPME fibres were inserted into the headspace and transferred to a 55 °C water bath for one hour, stopping further growth and allowing adsorption of volatiles onto the fibre. Optical density of duplicate broths at each time point was measured at 550 nm and dilutions performed to keep the measurement in the optimum range for the spectrophotometer. Plate counts were performed on LB agar to give cell counts at these densities. After incubation, the fibres were analysed by GC-MS as described in 4.2. These results were used to gauge the optimum optical density at which to sample *E. coli* volatiles by SPME.

Following this, a selection of *E. coli* isolates were obtained from the University of Bristol, consisting of four wild type CTX-M ESBL positive urinary isolates from the community [36] and four ESBL negative laboratory strains. Single colonies from fresh overnight cultures of each organism on LB agar at 37 °C were used to inoculate 250 mL of LB broth. These were then incubated overnight at 37 °C with shaking. 200 µL of each primary broth was then transferred to 2 × 10 mL of LB broth in identical headspace vials. Turbidity of one of the vials was monitored by optical density at 550 nm until the required growth density was achieved. At this point the SPME fibre with Carboxen^®^/Polydimethylsiloxane (CAR/PDSM) coating was inserted through the septum into the headspace and further incubated for 1 hour at 55 °C as before. The fibres were then retracted into their holders and the ends sealed before being placed in a transportable icebox at −15 °C. When all fibres had been exposed, the box was transported to the gas chromatography-mass spectrometer (GC-MS) laboratory. Four biological replicates of each strain were performed on different days from fresh primary agar culture each time.

### 3.2. VOC Analysis

After the extraction and transport, the fibers were immediately introduced into a gas chromatography injector port with 0.75 mm ID splitless glass liner (PerkinElmer, Inc., Waltham, MA, USA), set to splitless mode and a temperature of 240 °C. The thermal desorption of VOCs was carried out for 10 min to avoid carryover (data not presented). Analysis of VOCs was performed using a Perkin Elmer GC-MS Clarus 500 instrument (PerkinElmer, Inc., Waltham, MA, USA). The separation of VOCs was undertaken using a Zebron-624 column; 60 m × 0.25 mm × 1.40 μm, (Phenomenex, Torrance, CA, USA). The carrier gas was 99.9995% pure helium (AirProducts, Crewe, UK) at a constant flow of 1.5 mL/min. The oven temperature program was set as follows: 40 °C hold for 2 min, and increase to 240 °C at a rate of 10 °C/min and then maintained at the final temperature for 8 min, giving a total run time of 30 min. Mass spectra were obtained by electron ionisation acquired in full scan mode with a scan range of m/z 35–450 used for data acquisition. The operating conditions for the mass spectrometer were as follows: electron ionisation mode at an energy of 70 eV; transfer line and ion source temperatures were 280 °C and 180 °C, respectively. Total ion chromatograms (TIC) were analysed with the Turbomass software (PerkinElmer, Inc., Waltham, MA, USA) and compounds were expressed as the area under the curve. For the identification, the peaks were deconvoluted using the free Automated Mass Spectral Deconvolution and Identification System (AMDIS) software by the National Institute of Standards and Technology (NIST, Gaithersburg, MD, USA) and tentatively identified by comparison of the mass spectra with the NIST/EPA/NIH Mass Spectral Library (version 2.2, 2014, Gaithersburg, MD, USA). Only the components with a match factor >80% were listed by name in the Tables. Moreover, retention indexes for the detected compounds were calculated according to d’Acampora Zellner and co-authors [37]

### 3.3. Data Analysis

Comparison of the VOC profiles of ESBL positive and negative strains was performed at each retention time using the Mann−Whitney test. The peak areas of VOCs which significantly differed between sensitive and ESBL-producing strains were used in a multivariate assessment using linear discriminant analysis. A square root variance stabilising transformation of peak areas was undertaken prior to multivariate assessment in order to produce a robust model not prone to the presence of outliers. Leave-one-out cross-validation was used in the assessment of predictive accuracy.

## 4. Conclusions

The concept of rapidly detecting antibiotic-resistant bacteria by analysing VOCs is ambitious, but our results and those of others suggest the approach is promising. Differences in ESBL-positive and -negative isolates were rapidly distinguished by SPME with GC-MS in under 2 h, which compares well with 24 h or more using routine methods. Statistically significant differences were found between the VOC profiles of CTX-M-15 positive and negative bacteria. The study of VOCs and linking specific VOCs to certain antibiotic strains may help elucidate a better understanding of bacteria−host interactions in addition to allowing specific detection at earlier time points compared to conventional methods.

The next stage of this study will be to expand the VOC profile testing of ESBL strains of *E. coli* such as those carrying other CTX-M variants or those producing AmpC enzymes. It is also important to test strains with non-beta-lactam resistance profiles. Following this, attempts can be made to accurately categorise novel strains in a fully blinded study. Furthermore, with more complete identification of VOCs of note, it may be possible to develop specific enrichment media to allow direct examination urine samples as well as the sensor-based devices dedicated to the individual VOCs that can be used in a medical, point-of-care practice.

## Figures and Tables

**Table 1:** List of the VOCs detected in the headspace above the *E. coli* culture at 300 min.

Retention Time	Tentatively Identified VOCs	RI ^1^
6.99	unknown 1	419
7.14	unknown 2	428
9.84	Ethanol	590
10.44	Acetone	626
10.86	unknown 3	652
11.70	Propanol	702
12.01	unknown 4	721
12.36	2-butanone	742
13.04	Isobutanol	782
13.17	unknown 5	790
13.34	unknown 6	800
13.44	Isopentanal	806
13.77	1-butanol	826
14.06	unknown 7	844
14.15	Dimethylfuran	849
14.67	unknown 8	880
14.90	propanoic acid	894
15.12	1-pentanol	907
15.24	DMDS	914
15.47	Toluene	928
15.71	unknown 9	943
16.19	Dimethylheptene	971
16.44	Octane	986
18.24	Methylpyrazine	1094
18.90	2,2,4,6,6-pentamethylheptane	1134
19.79	Benzaldehyde	1187
26.76	Indole	1606

^1^ RI—retention index.

**Table 2:** List of tentatively identified VOCs detected in 84 samples of bacterial cultures.

Retention Time (mins)	Tentatively Identified VOCs	RI ^1^	*p*-value
9.91	ethanol	595	0.112
10.09	unknown 3	605	0.740
10.5	acetone	630	0.501
10.65	dimethyl sulfide	639	0.756
11.56	isobutanal	694	0.892
11.64	unknown 10	698	0.193
11.8	propanol	708	0.650
12.27	butanal	736	0.057
12.43	2-butanone	746	0.851
12.86	unknown 11	772	0.861
13.13	isobutanol	788	0.942
13.5	isopentanal	810	0.052
13.65	methylbutanal	819	0.760
13.71	pentanone	823	0.413
13.8	butanol	828	0.647
14.61	unknown 12	877	0.063
14.76	unknown 8	886	0.228
15.15	pentanol	909	0.124
15.33	DMDS	920	0.767
15.51	toluene	931	0.957
15.77	unknown 9	946	0.759
16.13	butyl acetate	968	0.117
16.25	unknown 13	975	0.317
16.43	methylbutenal	986	0.917
16.52	unknown 14	991	0.507
**16.68**	**butanoic acid**	1001	**0.040**
16.83	methylpyrazine	1010	0.660
17.03	unknown 15	1022	0.104
17.12	dimethylheptane	1027	0.314
17.19	1-methoxy-2-propyl acetate	1031	0.507
17.27	ethylbenzene	1036	0.410
17.38	x-xylene^2^	1043	0.925
17.55	allyl butyrate	1053	0.065
17.64	unknown 16	1058	0.246
17.79	butyl propionate	1067	0.069
17.87	unknown 17	1072	0.826
17.93	2-heptanone	1076	0.754
18.3	2,5-dimethylpyrazine	1098	0.713
18.38	unknown 18	1103	0.407
**18.5**	**unknown 19**	1110	**0.011**
18.77	methyl-heptanone	1126	0.069
18.8	unknown 20	1128	0.513
18.96	2,3,4,6,6-pentamethylheptane	1138	0.101
19.04	unknown 21	1142	0.304
19.11	unknown 22	1147	0.054
19.2	unknown 23	1152	0.025
19.26	butyl isobutyrate	1156	0.067
**19.47**	**unknown 24**	1168	**0.030**
19.62	unknown 25	1177	0.478
19.82	dimethyl trisulfide	1189	0.943
19.83	benzaldehyde	1190	0.203
19.92	D-limonene	1195	0.943
19.98	unknown 26	1199	0.224
20.24	2-ethyl-hexanol	1214	0.155
**20.4**	**unknown 27**	1224	**0.003**
20.48	unknown 28	1229	0.914
20.52	unknown 29	1231	0.814
20.57	unknown 30	1234	0.781
20.79	unknown 31	1247	0.200
20.91	butylglycol acetate	1255	0.098
20.94	unknown 32	1256	0.199
21.00	unknown 33	1260	0.264
21.21	benzyl alcohol	1273	0.536
21.29	benzyl alcohol	1277	0.088
21.39	unknown 34	1283	0.399
21.59	acetophenone	1295	0.211
21.71	unknown 35	1303	0.580
21.78	unknown 36	1307	0.878
21.89	unknown 37	1313	0.161
22.07	cresol	1324	0.197
22.79	unknown 38	1367	0.202
23.01	unknown 39	1381	0.837
23.39	unknown 40	1403	0.259
23.46	unknown 41	1408	0.822
23.9	pentyllfuran	1434	0.873
24.02	unknown 42	1441	0.461
24.18	unknown 43	1451	0.369
**24.36**	**2-dodecanone**	1462	**0.005**
24.57	unknown 44	1474	0.363
**25.07**	**unknown 45**	1504	**0.045**
26.48	unknown 46	1589	0.550
26.87	indole	1612	0.156

^1^ RI—retention index. ^2^ x means unknown isomer. The compounds selected to the multivariate assessment are presented in bold. The *p*-values after the comparison of the peak areas of sensitive and resistant bacteria using Mann-Whitney test.

## Supplementary Materials

The following are available online at https://www.mdpi.com/2079-6382/9/11/797/s1, Figure S1: The mass spectra of unknown compounds used for the distinguishing of resistant and sensitive strains.
