# Supplementary material for: Towards the Identification of Antibiotic-Resistant Bacteria Causing Urinary Tract Infections Using Volatile Organic Compounds Analysis—A Pilot Study
Source: Antibiotics (Basel). 2020 Nov 11;9(11):797. doi: 10.3390/antibiotics9110797 (PMC7697827; doi:10.3390/antibiotics9110797)

**Supplementary Figure A1.** The mass specta of unknown compounds used for the distinguishing of resistant and sensitive strain.


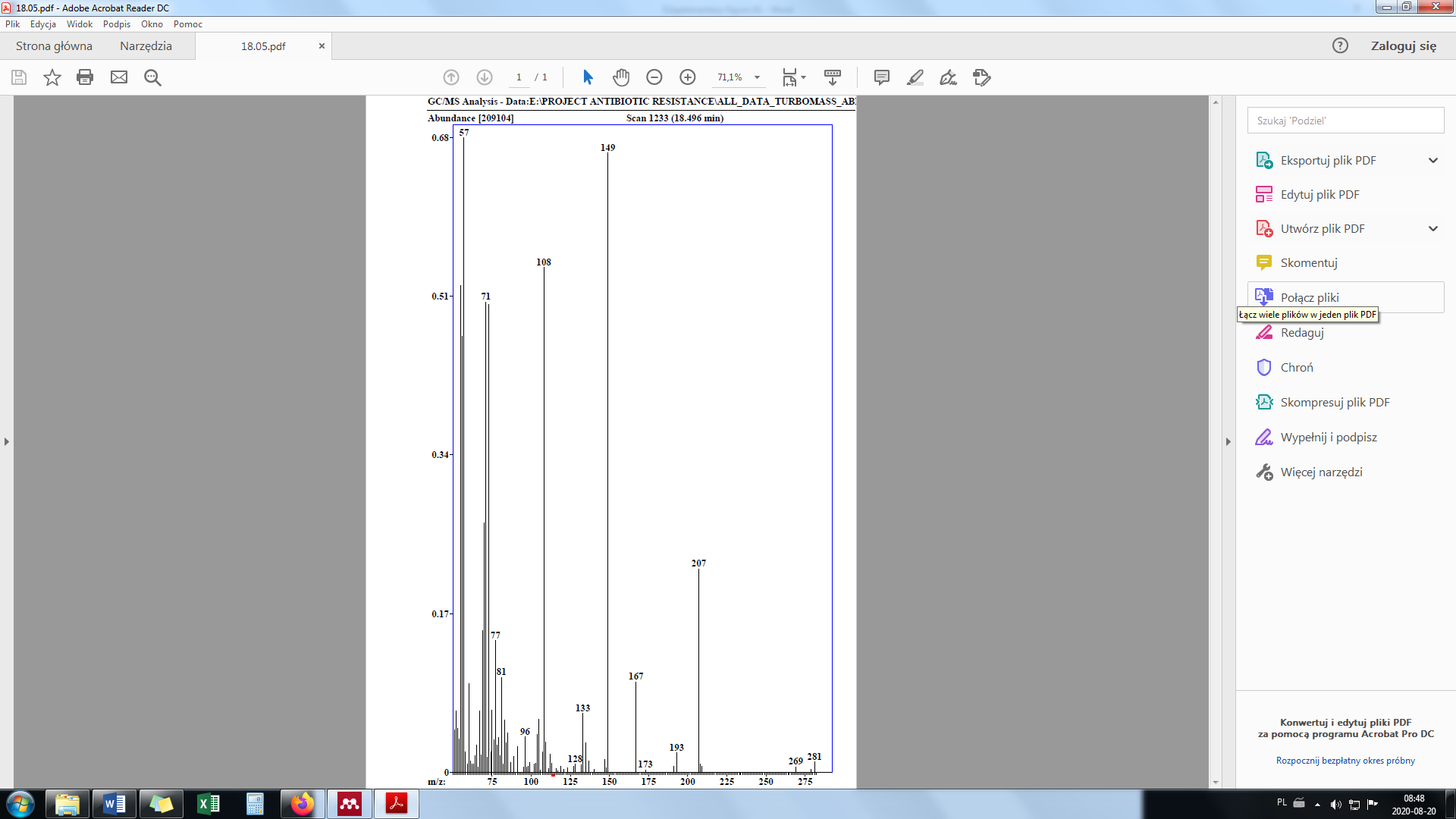


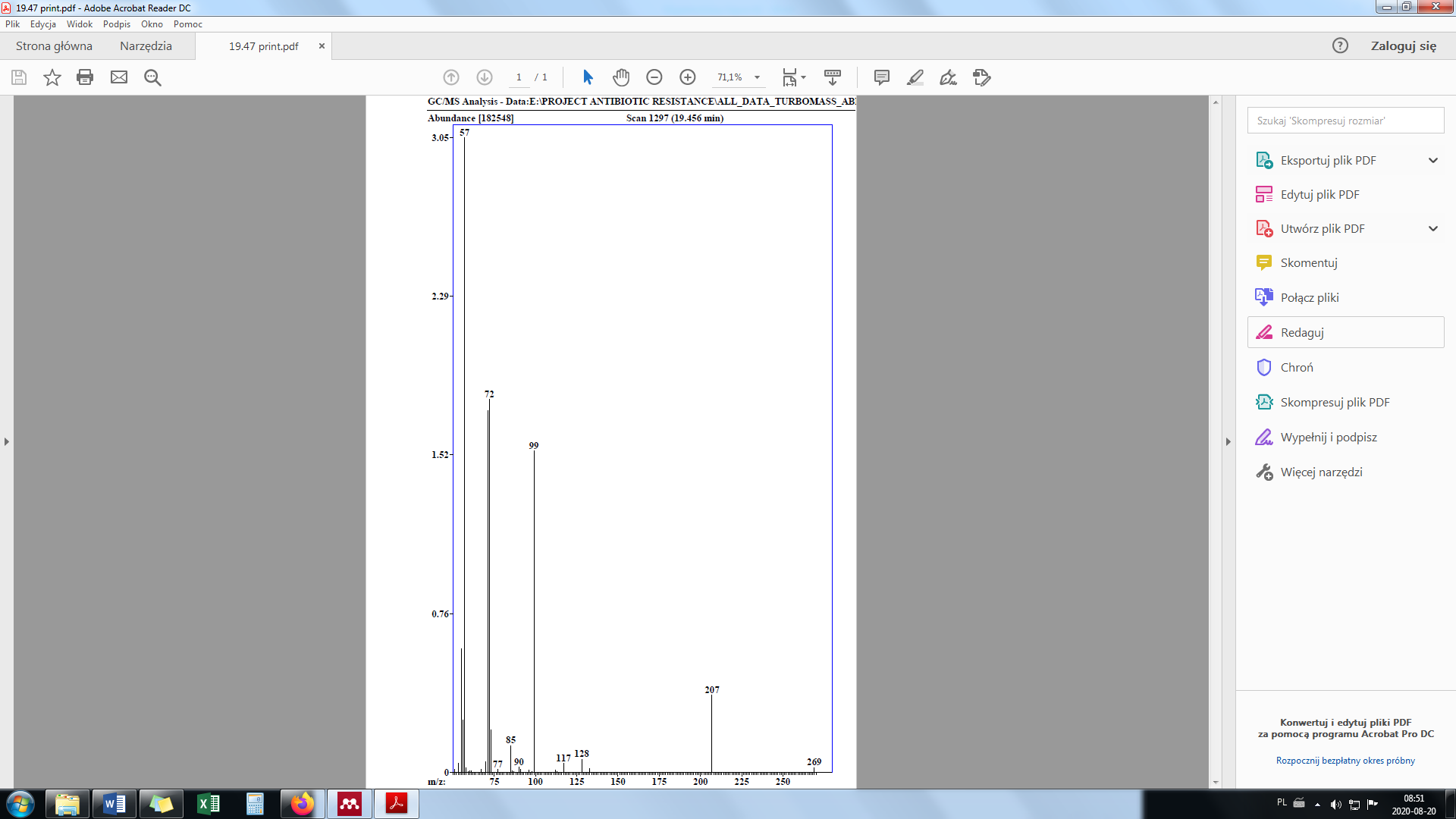


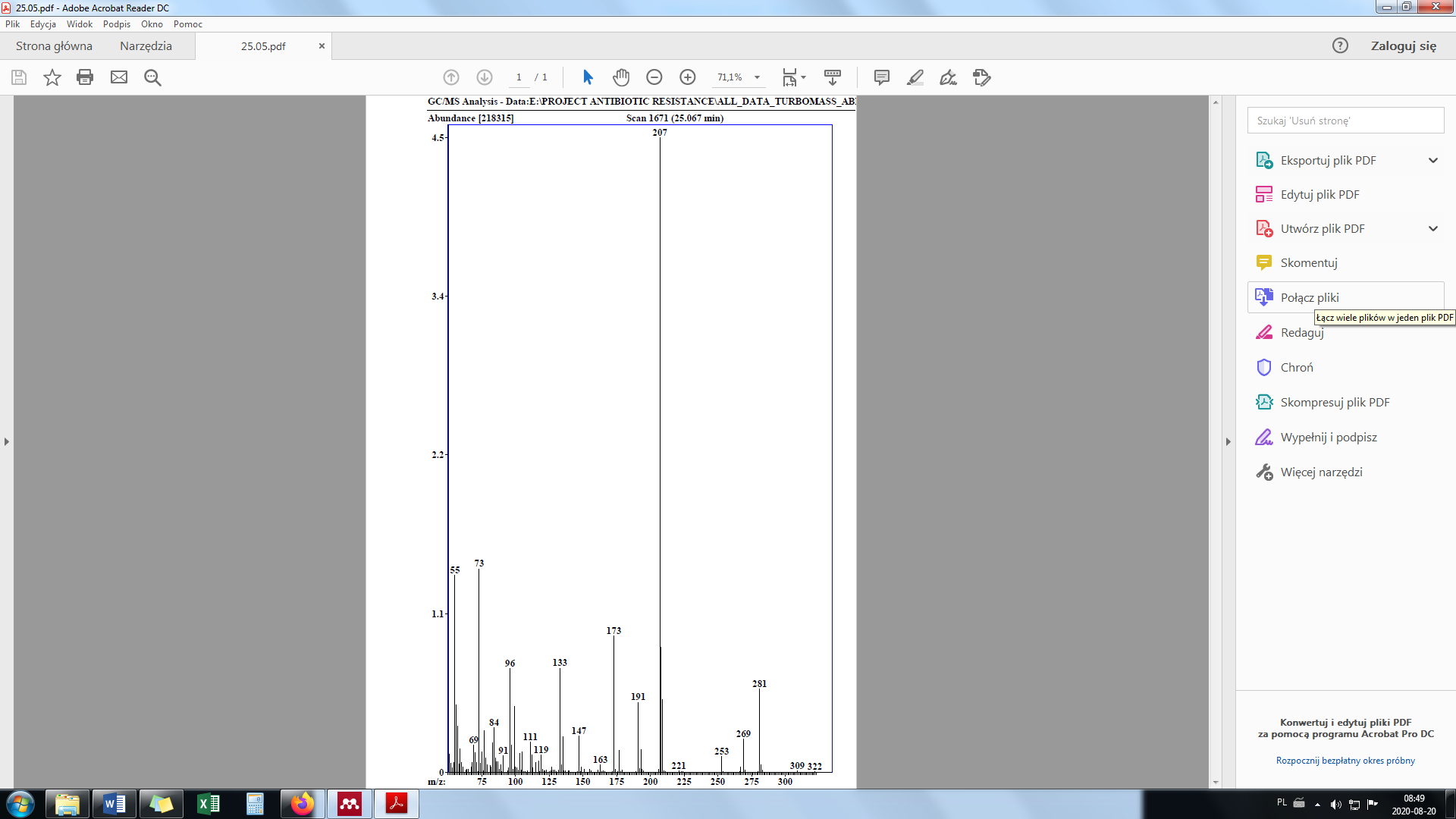


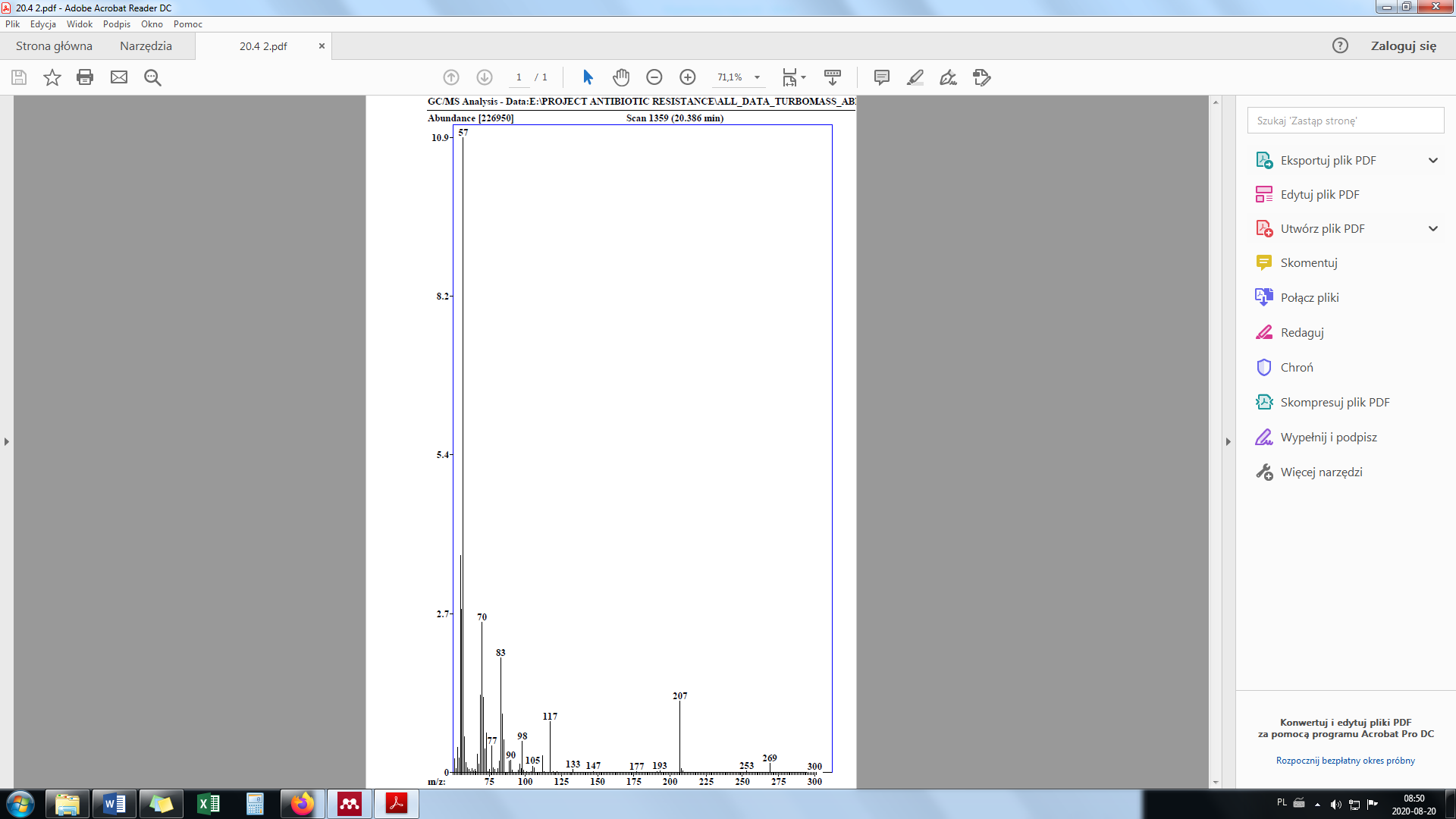

Supplement: Supplementary file 1 [file antibiotics-09-00797-s001.zip › SUpplementary figure A1.docx]
